# Supplementary material for: Pectin Remodeling and Involvement of AtPME3 in the Parasitic Plant–Plant Interaction, Phelipanche ramosa–Arabidospis thaliana
Source: Plants (Basel). 2024 Aug 5;13(15):2168. doi: 10.3390/plants13152168 (PMC11314565; doi:10.3390/plants13152168)
Supplement: Supplementary file 1 [file plants-13-02168-s001.zip › plants-3121017-supplementary.pdf]

Supplementary Materials

Table S1: Primer sequences of houskeeping (*APT1*, *TIP41* and *CLATHRIN)* and *PRE* genes used for molecular analyses.

Relative gene expressions of *PME* were measured using stably expressed reference genes (*APT1*, *TIP41* and *CLATHRIN*) with similar results. Only those obtained with the housekeeping gene *APT1* are shown in the paper.

Gene name	Forward primer sequence	Reverse primer sequence
TIP41 (At4g34270)	5'-GCTCATCGGTACGCTCTTTT-3'	5'-TCCATCAGTCAGAGGCTTCC-3'
APT1 (At1g27450	5'-GAGACATTTTGCGTGGGATT-3'	5'-ATTTTAAGTGGAACA-3'
CLATHRIN (At5g46630)	5'-GTTTGGGAGAAGAGCGGTTA-3'	5'-CTGATGTCACTGAACCTGAACTG-3'
PAE7 (At4g19410)	5'-ATGCGAGCATTGTCACCGGTTC -3'	5'-CCGACTGCTTTCGCAATTCTCG-3'
PME12 (At2g26440)	5'-ACTTTCCGATCTGCTACTCTTGCG-3'	5'-TAGTGCAACCGCTTGGTGCTTC-3'
PME17 (At2g45220)	5'-AGCAAGGCGTTTACAGTGAGAACC-3'	5'-CCGCGACAGTAGCTGAGTTAAAGG-3'
PME18 (At1g11580)	5'-TTTCCAATCGCTTCCCGTCGTG-3'	5'-TCGCGGTCACCTTAAGTGTCTTG-3'
PME20 (At2g47550)	5'-TGAGTGCTGCCATCACCAATGAG-3'	5'-CCTTCTTGGCACCCAACCTTTG-3'
PME31 (At3g29090)	5'-GGGAAACGCAGAGAACGAGAGAAG-3'	5'-ACCCGGACCAAAGCACCTATAC-3'
PME35 (At3g59010)	5'-CCGCCATGGGAGATGGATTCATAG-3'	5'-AGTTTGGTCCGGCACTGTTCAC-3'
PME40 (At4g02320)	5'-ACTTCAAGACCCGGTGGATGAG-3'	5'-AACCTGGTTTCACTCGAGTTGGG-3'
PME41 (At4g02330)	5'-TACATCGCCGAACTTCGTTGCC-3'	5'-GCTTCTCTGGTCCAGCGGTATTTC-3'
PME44 (At4g33220)	5'-CACTTTCCGATCAGCTACATTCGC-3'	5'-ATCTCAGTGCTACCGCCTGATG-3'
PME51 (At5g09760)	5'-GGTTCGTTAAACGCCGGTATGC-3'	5'-TCTCCTACCACTCCGACAGTTG-3'
PME59 (At5g51490)	5'-GGTCGGAGATGGAATGAGATCCAC-3'	5'-GATACCGGCAGTAGCGGAATTG-3'
PME62 (At5g55590)	5'-TTTACAGGGAGAAGGTGATTGTGC-3'	5'-TCTCCCGCATAGCTCTCATTACC-3'

Table S2: Expression analysis of *PAEs* genes (Mean ± SD) in *A. thaliana* roots (6 hai, 12 hai and 12 dai) in WT and *Atpme3-1*, infested or not. Target genes were normalized to the housekeeping gene *APT1* as internal control (n = 2 technical replicates per genotype and condition). nd = non-detectable. *PAE1* and *PAE12* are never detected in our conditions.

		PAE2	PAE3	PAE12	PAE4	PAE5	PAE7	PAE8	PAE9	PAE10	PAE11
6 HAI	*WT control*	0.00007 ± 0.00004	0,033 ± 0.007	0,159 ± 0.006	0,080 ± 0.022	0,235 ± 0.005	43,686 ± 4.003	0,0036 ± 0.0001	0,015 ± 0.002	0,0001 ± 0.00008	0,095 ± 0.017
	WT infested	0,00003 ± 0.00001	0,002 ± 0.0001	0,090 ± 0.004	0,012 ± 0.001	0,078 ± 0.009	15,703 ± 0.538	0,0003 ± 0.0002	0,002 ± 0.003	0,00002 ± 0.00001	0,020 ± 0.002
12 HAI	*WT control*	*nd*	0,040 ± 0.002	0,187 ± 0.013	*nd*	0,277 ± 0.017	41,274 ± 3.246	*nd*	0,006 ± 0.005	0,0002 ± 0.00003	0,109 ± 0.016
	WT infested	*nd*	0,009 ± 0.001	0,082 ± 0.006	*nd*	0,071 ± 0.005	14,228 ± 0.602	*nd*	0,003 ± 0.002	0,00003 ± 0.00001	0,033 ± 0.0004
14 DAI	*WT control*	*nd*	*nd*	*nd*	*nd*	*nd*	76,291 ± 3.380	0,022 ± 0.004	0,022 ± 0.010	*nd*	0,089 ± 0.012
	WT infested	*nd*	*nd*	*nd*	*nd*	*nd*	75,923 ± 3.403	0,049 ± 0.011	0,008 ± 0.002	*nd*	0,024 ± 0.009
6 HAI	*pme3 control*	*nd*	0,022 ± 0.011	0,017 ± 0.004	*nd*	*nd*	55,096 ± 14.594	*nd*	*nd*	*nd*	0,058 ± 0.011
	pme3 infested	*nd*	0,015 ± 0.004	0,008 ± 0.002	*nd*	*nd*	30,360 ± 2.231	*nd*	*nd*	*nd*	0,028 ± 0.005
12 HAI	*pme3 control*	*nd*	0,002 ± 0.001	0,063 ± 0.004	0,003 ± 0.002	0,064 ± 0.004	12,050 ± 0.855	*nd*	*nd*	*nd*	0,013 ± 0.001
	pme3 infested	*nd*	0,014 ± 0.003	0,137 ± 0.009	0,056 ± 0.021	0,022 ± 0.008	25,349 ± 0.924	*nd*	*nd*	*nd*	0,032 ± 0.002
14 DAI	*pme3 control*	*nd*	*nd*	*nd*	*nd*	*nd*	18,651 ± 0.230	*nd*	*nd*	*nd*	0,016 ± 0.005
	pme3 infested	*nd*	*nd*	*nd*	*nd*	*nd*	26,815 ± 2.566	*nd*	*nd*	*nd*	0,029 ± 0.002
